# Domestication of self-splicing introns during eukaryogenesis: the rise of the complex spliceosomal machinery

**DOI:** 10.1186/s13062-017-0201-6

**Published:** 2017-12-01

**Authors:** Julian Vosseberg, Berend Snel

**Affiliations:** Theoretical Biology and Bioinformatics, Department of Biology, Utrecht University, Padualaan 8, 3584 CH Utrecht, The Netherlands

**Keywords:** Spliceosome, Splicing, Introns, Evolution of complexity, Eukaryogenesis

## Abstract

**ᅟ:**

The spliceosome is a eukaryote-specific complex that is essential for the removal of introns from pre-mRNA. It consists of five small nuclear RNAs (snRNAs) and over a hundred proteins, making it one of the most complex molecular machineries. Most of this complexity has emerged during eukaryogenesis, a period that is characterised by a drastic increase in cellular and genomic complexity. Although not fully resolved, recent findings have started to shed some light on how and why the spliceosome originated.

In this paper we review how the spliceosome has evolved and discuss its origin and subsequent evolution in light of different general hypotheses on the evolution of complexity. Comparative analyses have established that the catalytic core of this ribonucleoprotein (RNP) complex, as well as the spliceosomal introns, evolved from self-splicing group II introns. Most snRNAs evolved from intron fragments and the essential Prp8 protein originated from the protein that is encoded by group II introns. Proteins that functioned in other RNA processes were added to this core and extensive duplications of these proteins substantially increased the complexity of the spliceosome prior to the eukaryotic diversification. The splicing machinery became even more complex in animals and plants, yet was simplified in eukaryotes with streamlined genomes. Apparently, the spliceosome did not evolve its complexity gradually, but in rapid bursts, followed by stagnation or even simplification. We argue that although both adaptive and neutral evolution have been involved in the evolution of the spliceosome, especially the latter was responsible for the emergence of an enormously complex eukaryotic splicing machinery from simple self-splicing sequences.

**Reviewers:**

This article was reviewed by W. Ford Doolittle, Eugene V. Koonin and Vivek Anantharaman.

## Background

Eukaryotic genes are in general composed of coding sequences interspersed by non-coding parts, the introns. Only after removal of these introns and splicing of the exons, a functional protein can be synthesised. The splicing reaction requires one of the most complex machines in the eukaryotic cell, the spliceosome, which consists of five snRNA molecules and over a hundred proteins [1, 2]. Two types of spliceosomes are present across eukaryotes, namely the major and the minor spliceosome. Each spliceosome splices its own type of introns, the U2-type introns for the major spliceosome and the U12-type introns for the minor counterpart.

The spliceosome is one of the numerous complex characteristics that emerged during eukaryogenesis. Eukaryotes are considered far more complex than prokaryotes, because of these evolved characteristics such as their larger genomes, cell sizes and intracellular compartmentalisation. However, some complex eukaryote-like features, such as large cells and internal membranes, have been observed in certain prokaryotes and some eukaryotes are less complex in organisation, cautioning for a too eukaryocentric view on complexity [3]. It has been firmly demonstrated that eukaryotes originated from the merger of two prokaryotes [4], an archaeal host related to the recently discovered Asgard phyla [5, 6] and a bacterial endosymbiont related to the Alphaproteobacteria. Lane and Martin [7] have proposed that the increased complexity of eukaryotes could solely be enabled by the surplus of energy provided by the mitochondrial endosymbionts, but their reasoning is challenged [3, 8]. The precise role of the mitochondria in the evolution of eukaryotic complexity remains therefore under lively debate.

The greater complexity of eukaryotes is additionally observed in the complexity of molecular machines, both for machines that are also present in prokaryotes (e.g., the ribosome and respiration chain complexes) [9–11] and eukaryote-specific complexes other than the spliceosome. The evolution of these molecular machines in their cellular context is within the scope of the emerging field of evolutionary cell biology [12–14]. One of the questions in this field is how the complexity of these complexes has evolved. For a complete understanding of the evolution of a complex, not only the intermediate steps have to be described, but also the evolutionary forces driving these steps. Multiple models have been proposed, emphasising the adaptive [11], neutral [10, 15–17] or maladaptive [18, 19] nature of additional components or interactions. Moreover, according to the biphasic model an increase in complexity is followed by a period of reductive evolution [20, 21].

Many steps were needed for the emergence of the complex spliceosome in the last eukaryotic common ancestor (LECA). The aim of this review is to reconstruct these steps and the subsequent changes in the complexity of the spliceosome in the distinct eukaryotic lineages, which is important for understanding why and how the complexity of this machine has evolved. This could additionally provide more insight into the evolution of other complex molecular machines. In this review we will focus on the snRNAs and main proteins of the major spliceosome.

### LECA’s spliceosomes

To separate the evolution of the spliceosome during eukaryogenesis from its evolution after the eukaryotic diversification, the spliceosome of LECA has to be reconstructed. The presence of spliceosomal components in all major eukaryotic lineages has revealed that LECA already had a complex major spliceosome, with five snRNAs and around eighty proteins [22]. Therefore, LECA’s spliceosomes would likely not be much unlike typical contemporary spliceosomes.

It has become clear that this complex spliceosome had to remove numerous introns from LECA’s transcripts. Multiple approaches have been followed for reconstructing the introns in LECA (reviewed in [23, 24]). The most sophisticated model used to date inferred an intron density of 4.3 introns per kilobase in LECA’s genome, which is only a fraction lower than the typical intron density of animal and plant genomes, but much higher than that of most protists [25]. Apparently, the complex nature of LECA’s spliceosome corresponded with its intron-rich genome.

The probable function of LECA’s spliceosomes can be inferred from experimental research on present-day spliceosomes, most of which has been performed in yeast, animals and plants. The main components of LECA’s major (U2-type) spliceosome were the five small nuclear ribonucleoproteins (snRNPs), consisting of snRNAs and dozens of other associated proteins [22] (composition and function of present-day spliceosomes reviewed in [1, 2, 26]). The uridine (U)-rich snRNAs in the spliceosome were U1, U2, U4, U5 and U6, each giving the accompanying snRNP its name. The snRNAs were tightly associated with a ring of either Lsm proteins (U6 snRNA) or Sm proteins (other snRNAs).

Rearrangements during the splicing cycle are crucial for spliceosomal functioning and these conformational changes were in LECA already effected by ATP-dependent RNA helicases. The precise composition of the spliceosome depended on the step in the splicing cycle. For example, U2, U5 and U6 snRNPs were present in the catalytically active spliceosome, whereas U1 and U4 snRNPs dissociated before the splicing reaction, as these were involved in splice site recognition and inhibiting U6 snRNA, respectively. The important regulatory serine/arginine-rich (SR) proteins and heterogeneous nuclear RNPs (hnRNPs), present across eukaryotes [27], were involved in exon and intron recognition and thereby splicing out the proper introns and enabling alternative splicing [1, 2, 26, 28]. After recognition of the 5′ and 3′ splice sites and the adenosine branch point nucleotide the first splicing step could be executed. In this transesterification reaction a nucleophilic attack created a covalent bond between the 5′ splice site and the 2′ OH group of the bulged adenosine, resulting in a lariat. In the following second reaction the exon ends were joined together and the lariat intron was released. In essence, LECA’s major spliceosome would likely not have been fundamentally different in composition and function from its present-day counterparts.

#### *Minor spliceosome and spliced-leader* trans*-splicing*

Although some earlier studies suggested otherwise [22, 27], additio ceosome evolved early in eukaryotes as well and was probably present in LECA [29, 30]. The minor spliceosome consists of its own specific snRNPs – U12, U22, U4atac and U6atac – which are functionally analogous to their major-spliceosomal counterparts, and U5 snRNP, which is shared between both spliceosomes [31]. The associated proteins in the minor spliceosome can be either specific to this complex or shared with the major spliceosome [31]. As mentioned before, the minor spliceosome excises a different kind of introns, the U12-type introns. These introns comprise only a small fraction compared with the U2-type introns in the organisms that contain both kinds of introns [31–33].

Most snRNPs of the major spliceosome are also involved in another related splicing reaction called spliced-leader (SL) *trans*-splicing, in which the SL RNA, which is carried by the SL snRNP, donates the first “exon” to the mRNA. This splicing mechanism is especially prevalent in some protist lineages, where it in some cases may account for all splicing events [34]. Based on its patchy presence pattern across eukaryotes it was initially proposed to have been present in LECA and subsequently lost multiple times in many lineages [22]. However, the observed pattern may also result from independent gain events due to horizontal gene transfer (HGT) [35] or recurrent mutational acquisition of SL RNA [35–37]. Whether the major spliceosome of LECA performed SL *trans*-splicing can therefore not unambiguously be established.

### Origin of the spliceosome

LECA likely already possessed two spliceosome types to process two different kinds of introns. These spliceosomes were approximately as complex as the ones typically observed in present-day eukaryotes. This poses the question how the complex spliceosome evolved during eukaryogenesis. Where did the proteins come from, how were they recruited into the spliceosome and what functions did their prokaryotic homologues, if present, execute?

The function of the spliceosome is removing introns from pre-mRNA molecules. The question how the spliceosome originated cannot be decoupled from the origin of the introns they remove. Without introns the spliceosome would be functionless and without the spliceosome the introns would cause the production of aberrant proteins. Different hypotheses have been proposed for the origin of spliceosomal introns. These will shortly be discussed before we turn to the origin of the spliceosome. Both the spliceosomal core and the introns themselves are likely derived from the very same origin, namely self-splicing introns.

#### Spliceosomal introns

The similarities between spliceosomal introns and group II self-splicing introns have been recognised for a long time. The latter are present in prokaryotes and in eukaryotic organelles. In mitochondria and plastids these introns are bona fide introns that lost their mobility potential, whereas in prokaryotes they are more properly regarded as retroelements [38, 39]. Group II introns (reviewed in e.g. [39, 40]) typically have a length of around 2–3 kb and consist of six RNA domains. The large domain I functions as a scaffold and recognises and positions the exons [41, 42], domains II and III enhance splicing catalysis [43] and domain VI contains the adenosine residue that functions as branch point [44]. Domain V is the most conserved domain and contains the catalytic triad, which binds the two catalytic divalent metal ions [43, 45, 46]. Domain IV is the largest, as it encodes a protein, aptly named intron-encoded protein (IEP). The maturase function of this versatile protein is required for the proper folding of group II introns, promoting RNA recognition and splicing [47, 48]. Moreover, its reverse transcriptase activity enables reverse splicing, which results in the proliferation of the introns in the host genome [47, 49].

There is an overwhelming amount of evidence supporting the homology between spliceosomal introns and group II self-splicing introns. The splice site recognition, branching mechanism, stereochemical course of the splicing reaction and the presence of similar RNA domain structures and a homologue of the IEP in the spliceosome (see below) demonstrate the similarities between the two intron types [39, 40, 50, 51]. Moreover, there is a known example of a group II intron that was transferred from mitochondria to the nucleus in a plant family and subsequently evolved into a spliceosomal intron [52], which underlines the evolutionary relationship between group II and spliceosomal introns.

Since group II introns are especially abundant in alphaproteobacteria and present in certain mitochondria, the most accepted view, first suggested by Cavalier-Smith in 1991 [53], is that spliceosomal introns originated from the alphaproteobacterial endosymbiont by endosymbiotic gene transfer (EGT) that later evolved into the mitochondria [39]. However, these self-splicing elements are also present in some archaeal lineages, including the Asgardian loki- and heimdallarchaeal lineages [5, 6], suggesting that they also could have been present in the archaeal host. In this context it is noteworthy that many bacterial genes in eukaryotes, proposed to have been acquired upon mitochondrial endosymbiosis [54], had more likely been acquired by the archaeal host before [55, 56]. Another hypothesis that was put forward but has fallen out of favour stated that the two kinds of introns share a common ancestor in the last universal common ancestor and originated from a kind of ‘protospliceosome’ in the RNA world [57, 58]. This hypothesis is related to the introns-early hypothesis, which postulated that protein-coding genes interspersed with introns were the ancestral state [59]. However, since it has been established that eukaryotes arose from within the Archaea [4–6], it is extremely unlikely that the introns were lost in the bacterial and all non-eukaryotic archaeal lineages, but remained present in the direct line leading to the eukaryotes.

As demonstrated by relatively recent intron gains, not all spliceosomal introns in present-day eukaryotes are derived from group II introns. These introns have an endogenous origin and different sources have been suggested, such as transposable elements, internal gene duplications and intronisation of translatable sequences [60]. Although it has been proposed, based on these recent intron gains, that spliceosomal introns in general had an endogenous origin [60], one should note that the origin of novel introns does not necessarily reflect the origin of the first spliceosomal introns. Given the evidence supporting a relationship with group II introns, an endogenous origin of spliceosomal introns during eukaryogenesis seems very unlikely.

#### Remnants of group II introns: snRNAs and Prp8

**Fig. 1:**
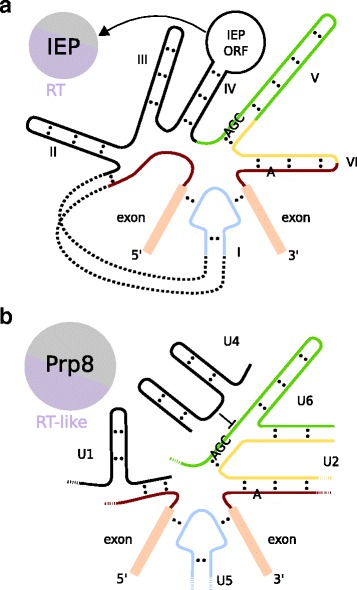
Resemblance between group II introns, and spliceosomal introns and snRNAs. **a** Simplified secondary structure of a group II intron (IIA) with its intron-encoded protein (IEP). The largest part of domain I has been omitted. The catalytic triad and adenosine branch point are explicitly depicted. The structures are coloured based on their similarity to spliceosomal structures (**b**). The black RNA domains do not have homologous structures in the spliceosome. **b** Simplified secondary structure of a spliceosomal intron with the snRNAs and Prp8. U1 and U4 snRNA are not homologous to group II intron domains

Numerous studies have noted the striking similarities in function and structure between the snRNAs and the group II intron domains and especially U6 snRNA and domain V look very similar (Fig. 1) [39, 40, 51]. For example, the catalytic triad and bulge are present in both structures, both bind divalent metal ions and they are functionally interchangeable [40, 51, 61, 62]. Parts of U5 snRNA, which is involved in exon recognition, resemble exon-binding sites in domain I and these parts are functionally interchangeable as well [50, 63–65]. Also domain VI and U2 snRNA show similarities [39]. The parallels between snRNAs and group II introns have led to the idea that the snRNAs are five pieces of a group II intron [66]. However, since the U1 and U4 snRNAs lack a clear similarity to group II domains, these probably have a different origin [39]. Remarkably, in some organelles group II introns are present in pieces, but splicing occurs normally [39, 67]. Furthermore, the experimental fragmentation of a group II intron in *Lactococcus lactis* demonstrated the potential for *trans*-splicing [68]. These observations make the hypothesised origin of the snRNAs from group II intron fragments plausible.

As mentioned above a group II intron usually encodes an IEP. A homologous protein of IEP functions in the spliceosome, namely pre-mRNA processing protein 8 (Prp8), which is present in the U5 snRNP. Prp8 is present in the spliceosomal catalytic core and likely functions as an assembly platform [50, 69, 70]. It is the largest and most conserved spliceosomal protein and interacts with the U2 and U6 snRNPs and especially the helicase Brr2 and GTPase Snu114, which are present in the U5 snRNP as well [1, 2, 71–74]. The first indication for the homology between IEP and Prp8 was the presence of a reverse transcriptase (RT)-like domain in Prp8, which is similar to the RT domain in IEP [75–77]. IEP did not only give rise to Prp8, but also to telomerase and the RT of non-long terminal repeat retrotransposons [76]. At some point Prp8 must have lost its RT activity [75, 78], thereby losing the ability for retromobility while maintaining its maturase function, which has occurred frequently for IEPs in organelles as well [39].

Group II introns can be classified based on RNA structures or phylogenetic groupings of IEP [39, 79–81]. The exon recognition in spliceosomal introns is more similar to the A subtype of group II introns [39]. It is not known how Prp8 and its paralogues relate to the different IEP groups, which could be informative for the source of the group II introns that evolved into the spliceosomal introns.

#### Sm and Lsm proteins

Each snRNA in the spliceosome is accompanied by a heteroheptameric ring consisting of either Sm or Lsm proteins, which are both members of the Sm family of proteins. For U6 snRNA it is an Lsm ring made up of Lsm2–8, whereas the ring surrounding the other snRNAs consists of SmB, −D3, −G, −E, −F, −D2 and –D1 [1, 22, 82–85]. The rings function as scaffolds, enabling interactions between the snRNAs and snRNP proteins, and they are specifically involved in snRNP biogenesis [86]. The central pore of the ring binds to uridine-rich stretches of RNA [83, 85]. The Sm rings remain stably attached to the snRNA, whereas the Lsm ring disassociates from the U6 snRNA, together with the other U6 snRNP proteins [2]. This dissociation is essential for the formation of the catalytic core [87]. U6 snRNA is also unique in the sense that its transcription is performed by RNA polymerase III instead of II, that it receives another 5′ cap, and is not exported to the cytoplasm [2, 88]. The import into the nucleus of the other snRNAs is dependent on their interaction with the Sm ring, which is assembled around the snRNA in the cytosol [26, 85].

Homologues of Sm and Lsm proteins are present in both bacteria and archaea. The bacterial homologue, Hfq, is encoded by a single-copy gene [86]. Hfq proteins comprise a homohexameric ring that functions as a RNA chaperone in multiple processes, for instance by mediating inhibiting interactions between non-coding RNAs and target mRNAs [86]. Archaea have between one and three Sm-like archaeal proteins, making homohexameric or homoheptameric rings, and despite many studies focussing on the structure of these proteins, their function is not well-characterised [86].

**Fig. 2:**
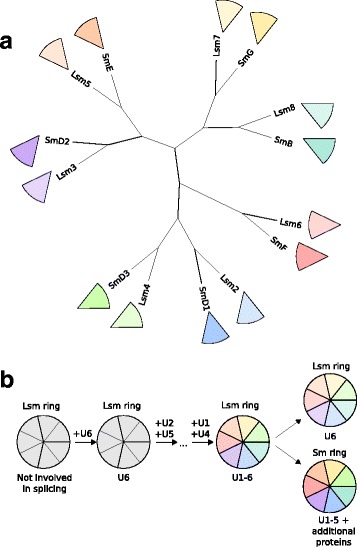
Evolution of the Sm and Lsm rings. **a** Tree depicting the scenario on the evolution of the spliceosomal Lsm and Sm proteins, as proposed in [90]. **b** Possible scenario for the evolution of the Lsm and Sm rings. A homoheptameric Lsm ring interacted with the *trans*-acting U6 snRNA, thereby facilitating splicing of degenerating self-splicing introns. While the Lsm ring became heteromeric upon duplication and subfunctionalisation of the Lsm protein, the *trans*-acting U2 and U5, which all originated from the introns, and U1 and U4 snRNAs formed RNP complexes with the Lsm ring. Upon duplication of the ring, U6 snRNA was bound by the Lsm ring, whereas the other snRNAs formed a complex with the newly formed Sm ring, followed by the addition of other proteins

Although an earlier study was unable to confidently infer the deep phylogenetic relationship between the eukaryotic Sm and Lsm genes [89], a more sophisticated analysis found that each spliceosomal Lsm gene was paired with an Sm gene (Fig. 2a) [90]. In both studies the relationship with the prokaryotic outgroup was inconclusive. It was suggested that because of the greater divergence of the Sm genes these had acquired a new function in forming the Sm ring, whereas the Lsm ring was the ancestral one. This would be consistent with the observation that Lsm rings are also involved in other processes, whereas Sm rings are specific to the spliceosome [89, 90]. Based on this, two waves of duplications were proposed, the first one leading to the seven spliceosomal Lsm genes and then duplication of each Lsm gene to an Lsm–Sm pair. The pairing was confirmed by the observation that several of the pairs have an intron at the same position when intron locations are mapped onto the alignments of these pairs across 22 species. A small number of introns are even shared between certain Lsm-Sm pairs, i.e. Lsm6 and Lsm8 share an identical intron position, as do Lsm3 and SmE. This is not trivial, as it implies that splicing could already take place before the early diversification of the Sm family in eukaryotes. Although the shared introns could reflect independent intron gain events, this is less likely since it is the case for multiple pairs and the inferred shared introns are present in multiple species. Furthermore, given the overall low number of introns (<3%) shared between paralogues originating from gene duplications during eukaryogenesis [91] and the high inferred number of introns shared between orthologues in LECA [92], this would suggest that these duplications occurred relatively late during eukaryogenesis.

Presumably, it started with a homoheptameric flexible Lsm-like ring (Fig. 2b). A first wave of duplications resulted in an Lsm heteroheptamer. Before these duplications splicing already took place and the Lsm ring might already have had a function in splicing. The specific steps from a homomeric to a heteromeric ring are difficult to infer. It has been suggested that once there was a heteromeric ring consisting of two different components, the heptameric nature of the structure accelerated the transition to an entirely heteromeric ring with seven different subunits [89]. The reason behind this is that seven is a prime, so the most stable heteromeric ring may be a completely heteromeric one. The resulting heteromeric nature of the ring enabled the steric specificity that is now present in these rings [89]. Duplication of the entire ring resulted in the more stable Sm ring, which became associated with all snRNAs but U6. It has been proposed that the origin of the nucleus resulted in this separation between U6 and the other snRNAs, due to the latter’s export out and subsequent Sm-mediated import into the nucleus [90].

#### Helicases, Snu114 and SR proteins: Addition of proteins involved in translation and RNA degradation

The ATP-dependent RNA helicases in the spliceosome are mainly from three families within the SFII superfamily, which is especially predominant in eukaryotes [93]. One of these is the eIF4A-DeaD family, which has in general only one representative in prokaryotes, DeaD, while in eukaryotes the family has vastly expanded to include around thirty distinct members, most of them functioning in the splicing reaction [93]. Eukaryotic eIF4A can be regarded as the equivalent of prokaryotic DeaD, because of their similar function in translation regulation [93]. The U5 snRNP-specific protein Brr2 is part of the Ski2p-LHR family within the SFII superfamily, whose members typically function in the exosome [93].

Another protein in U5 snRNP is the aforementioned GTPase Snu114, which interacts with Brr2 and Prp8 and is located near the catalytic site. Snu114 was already present in LECA [22] and is homologous to the ribosomal translocase EF-2 [94]. Apparently, multiple proteins involved in RNA degradation and translation were recruited into the spliceosome.

The SR splicing regulator proteins are characterised by an RNA recognition motif, which is also present in multiple prokaryotes, especially cyanobacteria [95]. A phylogeny based on these motifs pointed to a single origin for SR proteins as a sister group to the SR-like atypical RNPS1/SR45 proteins, albeit with marginal support [95]. Moreover, the radiation into three SR families and a family comprising the RNPS1/SR45 proteins likely had occurred before LECA. This example emphasises the importance of gene duplications in the origin of the spliceosome, as do the evolutionary histories of Sm proteins and helicases.

**Fig. 3:**
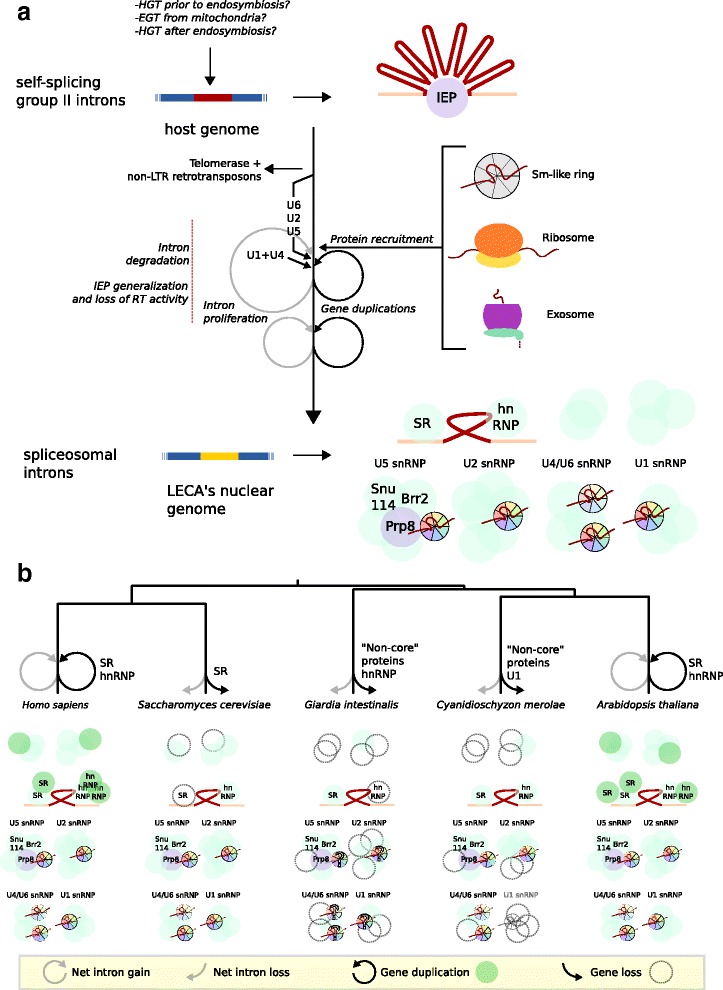
Evolution of the spliceosome. **a** Origin of the spliceosome during eukaryogenesis. The major steps resulting in the domestication of self-splicing introns in the early eukaryotes are depicted. **b** Subsequent evolution after eukaryogenesis resulting in the more complex or simple spliceosomes in five diverse eukaryotes. Besides the gain or loss of notable proteins the net loss or gain of introns is depicted for each lineage. The internal branches seemed to have experienced no large change of intron density [25]. The circles, except Snu114 and Brr2, represent an arbitrary number of proteins. The question marks in *Giardia*’s Lsm and Sm rings reflect the ambiguity about their exact composition [22, 90]

The evolutionary history of many other spliceosomal proteins has been clarified to a lesser extent. The exact source of each component, i.e. whether it was present in the archaeal host, the bacterial endosymbiont, was acquired later via HGT or was a unique eukaryotic invention, is obscure as well. The aforementioned examples demonstrate that duplicates of proteins active in other RNA processes in the first stages of eukaryogenesis supplemented the group II intron core in the emerging spliceosome. Subsequent expansions of these protein families resulting in many paralogues within the spliceosome contributed to the vast complexity of the machine (Fig. 3a).

#### Order of events

Several papers put forward a speculative order of events that led to the emergence of the spliceosome. The starting point for these scenarios is the presence of self-splicing group II introns, including their maturases, in the host genome. For example, Anantharaman et al. [93] proposed that the Sm proteins were recruited by the self-splicing introns as protein cofactors, followed by RNA helicases, of which some had an exosomal function. The subsequent partial degeneration of the introns resulted in the snRNAs that partially replaced the introns themselves in the splicing machinery. On the other hand, the scenario of Martin and Koonin [96] starts with the decay of self-splicing introns, requiring the recruitment of group II-derived RNAs, which evolved into the snRNAs, and associated Sm proteins. Subsequently, additional proteins were added to this spliceosomal core. The model of Veretnik et al. [90] also begins with RNA components, at least U6 snRNA, associated with a homomeric, and later heteromeric Lsm ring. The interaction between U6 snRNA and the Lsm ring could according to this scenario be seen as a ‘frozen event’. The addition of other snRNAs, which became later on accompanied by the Sm ring, was the next step. Other components were added to the spliceosome successively. These scenarios differ most in their proposed timing of the origin of snRNAs as distinct units. The models have in common that they regard Sm proteins as early additions to the spliceosome, as they are at the core of the complex.

The timing of the decay of self-splicing introns to spliceosomal ones, on the other hand, differs in these scenarios. Since group II introns have not been detected in nuclear genomes, all introns were apparently converted to spliceosomal introns or completely lost at some point before LECA. Complications with the expression of the targeted gene that arise when a group II intron is integrated in a nuclear gene were suggested to have caused their disappearance [97, 98]. However, their presence in non-coding regions would probably not have posed a challenge, implying that this cannot be a sufficient explanation [99]. Although the low intracellular Mg^2+^ concentration in eukaryotes may have posed a barrier to group II introns in eukaryotic genomes, including protein-coding genes [100], it does not seem an impossible barrier to overcome, especially given that splicing of group II introns can be induced in the cytosol in yeast [97, 98]. Therefore, a more complete and sufficient explanation remains to be postulated.

### Spliceosomal diversity after eukaryogenesis

Evidently, much research has focused on the many steps leading to the complex nature of the spliceosome in LECA. Nevertheless, the lack of access to intermediate stages poses a challenge to precisely reconstruct the evolution of the spliceosome. The wide diversity of eukaryotic spliceosomes provides a rich source of complementary data that show both further complexification as well as simplification of the spliceosome (Fig. 3b). The occurrence of these processes has implications for our understanding of the origin of the spliceosome.

#### Increase in complexity

In at least two lineages the spliceosome has become more complex. The most prominent complexification is the expansion of splicing regulator proteins, which are involved in the recognition of exons and introns, in plants and animals. The SR family has expanded in multicellular eukaryotes, especially in plants [27, 101]. Angiosperms have typically around twenty SR proteins, animals about ten and protists two or three [101]. Also the number of hnRNP proteins has increased in multicellular organisms, which is even more pronounced than the SR family expansion [102]. The greater hnRNP diversity is especially prominent in vertebrates, whose genomes encode between twenty and forty of these proteins [27]. Other animals and plants typically have between ten and fifteen hnRNPs, which is much more than the one hnRNP present in yeast [27, 102]. Furthermore, other regulatory factors, such as ELAV-like and CELF proteins and kinases that phosphorylate SR proteins, have expanded in vertebrates [27, 103]. The diversification of these sets of proteins had already occurred before the last common ancestor of metazoans and the subsequent expansion in vertebrates is proposed to originate from the whole-genome duplications [27, 103]. Genome duplications may account for the extensive SR family expansion in plants as well [101].

Although the high number of alternative splicing events in animals relative to other eukaryotes could be related to the expansion of the splicing regulator repertoire in these organisms [104], this is not evidently the case in plants [101]. It is believed that due to the increased SR and hnRNP repertoire non-optimal splice sites were tolerated, since purifying selection on splice site sequences was relaxed [102]. The differences in the splicing regulator repertoire might underlie the differential preference for exon skipping in animals, compared to intron retention in plants and other eukaryotes as alternative splicing mechanism [104].

#### Reduction in complexity

In many other eukaryotic lineages the evolution of the spliceosome is characterised by simplification. Both the loss of some subunits and the complete loss of the spliceosome have occurred. Based on draft genomes, introns and spliceosomal genes seem to be completely absent in a few microsporidia species [105, 106] and in a diplomonad species [107]. The complete nucleomorph genome of a cryptophyte species also demonstrated a complete loss of introns and spliceosomal RNAs and proteins [108].

In contrast with the aforementioned loss of both the major and minor spliceosome and corresponding introns, the loss of only the latter has been more common. The minor spliceosome is present in representatives of all eukaryotic supergroups, but has at least 9 times been lost during eukaryotic evolution [29, 30]. This loss is accompanied by a loss of U12-type introns, which can either be a complete loss of these introns or a conversion to U2-type introns [109]. The latter can be accomplished by mutations or a shift of the splice site, which were both found in the lineage leading to *Caenorhabditis elegans* [109]. Losses of U12-type introns are more frequently observed than conversions [33].

In addition to the complete loss of either the minor spliceosome or both types of spliceosomes, reduced spliceosomes have been observed and more thoroughly analysed in at least three lineages. This loss of spliceosomal subunits is associated with a lower number of introns [110–112]. Numerous proteins can be absent from these spliceosomes. For example, the classical SR proteins appear to have been lost in some lineages, including *Saccharomyces cerevisiae* [22, 102]. Even proteins that can be considered to belong to the core of the complex, like the snRNAs, Sm proteins and some other snRNP proteins, are not present in all eukaryotes. For instance, some organisms can perform splicing without a full set of Sm/Lsm proteins [22, 90]. The snRNAs of the diplomonad *Giardia lamblia* have characteristics of both major and minor spliceosomal snRNAs and therefore the reduced spliceosome of this organism is suggested to be a hybrid [111, 113]. Many spliceosomal proteins are missing in this diplomonad, but most U2 snRNP proteins and the core U5 snRNP proteins are still present [110]. A similar reduction pattern has been observed in the red alga *Cyanidioschyzon merolae* [112, 113]. The proteins remaining in both organisms correspond with the catalytic core of the spliceosome [113]. On top of that, *C. merolae* seems to perform splicing without a U1 snRNP, as both U1 snRNA and U1 snRNP-specific proteins appear to be missing [112]. This loss is hypothesised to mimic an ancestral state during eukaryogenesis in which U1 had not yet been added to the primordial spliceosome [113]. The observations that U1 snRNA does not have a clear analogue in group II introns and that it can be lost in the spliceosome, are arguments for a later addition of the U1 snRNP to the early spliceosome.

### Evolutionary models of spliceosomal evolution

Numerous scenarios for the evolution of the spliceosome have been suggested. Usually this concerns a description of what happened, but to truly comprehend the evolution of the spliceosome a transition has to be made from a mere description to addressing the evolutionary forces that shaped this complex machine. A number of hypotheses concerning these forces have been proposed, as mentioned in the introduction. They propose that either the addition or loss of each component of a complex is an adaptation or that solely neutral processes are responsible for the shifts in complexity.

#### Adaptive model

Since the establishment of the power of natural selection, adaptive explanations for biological observations have been the most prominent and widely accepted. Many biological papers propose an adaptive explanation for their observations, albeit often implicitly. Such explanations can in many cases be criticised as being just-so stories that lack proper evidence [114]. The role of natural selection in reductive evolution is widely established, but this is not the case for its role in the increase in complexity. In that case, each addition should have been selected for. The function of the spliceosome is clear, namely removing spliceosomal introns from pre-mRNAs. The large compositional complexity is believed to have arisen to make splicing more efficient and precise and to stabilise the complex [1, 11, 96, 114]. However, the spliceosome seems to perform worse in these respects compared to the self-splicing capacity of group II introns [17]. Furthermore, in many adaptive scenarios an innovation is necessary to compensate for a detrimental event, which is of course maladaptive, such as the evolution of snRNAs to compensate for degenerated introns and the higher complexity needed to cope with the expansion of introns into genes and the loss of clearly defined exon-intron boundaries [39, 93, 96]. Also a nucleus would be selected for due to the emergence of introns in genes, which resulted in the detrimental synthesis of aberrant proteins [96, 115]. Another proposed advantage of a complex spliceosome is that it enables better regulation called fine-tuning, which is especially the case in organisms that have extensive alternative splicing [11]. An issue related to the emergence of the spliceosome is the origin of spliceosomal introns. The main adaptive value of these sequences is proposed to be an expansion of the proteome by facilitating exon shuffling and alternative splicing [11]. This basically means that the increased genomic complexity due to introns is to enable an increase in complexity. Note that in all these adaptive scenarios the present-day function does not necessarily correspond to why the system originated in the first place [114]. In general, many adaptive roles for the spliceosome have been proposed, all giving reasons why splicing could be adaptive once you have it, yet failing to provide a reason for its very origin.

#### Neutral model

In the constructive neutral evolution model the increase in complexity can be seen as a ‘drunkard’s walk’ into the more complex possibilities of a system [15]. The concept of presuppression is central in this ‘walk’ [9]. This means that a certain factor (A) is bound by another factor (B), which does not affect the function of the former. The effects of mutations in factor A that would normally impair its function, are now suppressed by the interaction with factor B. These previously deleterious mutations are therefore now neutral and can become fixed in the population. This results in the dependence of factor A on factor B. In this way other mutations that strengthen this dependence may occur, resulting in a ratchet-like process. Reversal to the ancestral, independent state is possible, but given that there are more possibilities to increase this dependence, this is less likely. Via this mechanism of presuppression neutral evolution could result in a ratchet-like increase in complexity [10, 16].

A well-established example of a similar neutral process resulting in increased complexity is subfunctionalisation of paralogues after gene duplication. A combination of constructive neutral evolution and subfunctionalisation may explain the formation of a heteromeric protein complex from a homomeric state. Finnigan et al. [116] demonstrated this experimentally for the evolution of the fungal vacuolar H^+^-ATPase ring and suggested that this could have been the case in other multi-paralogue complexes as well. As the spliceosome comprises multiple paralogues, such as the Sm proteins and helicases [19, 90, 93], a similar mechanism might have been operating in its evolution towards greater complexity as well.

It should be noted that it is difficult to classify an increase in complexity as neutral. As pointed out by Lynch [18], each embellishment makes a biological system more susceptible to inactivation by mutations. The additional feature should either provide a direct advantage to become fixed in the population or selection should be inefficient to remove this variant due to the larger effect of genetic drift in case of a small effective population size [18]. The latter is believed to have been the case during eukaryogenesis and this may explain the many complex characteristics of eukaryotes, including complex machineries such as the spliceosome [18, 19, 38].

In a neutral scenario the spliceosome would have evolved from the addition of new RNAs and proteins that do not improve the efficacy of the splicing reaction to the catalytic core inherited from the group II intron ancestor. Moreover, at some point the structural RNA elements in the group II introns were replaced by fragments of other group II introns that acted as *trans*-acting RNAs. These primordial snRNAs and an IEP that acts as a general maturase, which does not only assist splicing of its own intron, would have made the RNA domains of the introns and a dedicated IEP redundant. In this way previously deleterious mutations in the introns are now presuppressed by the action of this *trans*-acting RNP complex, resulting in the loss of self-splicing features. This primordial spliceosome would also allow the spread of inactive group II introns and intronised sequences unrelated to group II introns in the genome. The already established nucleus would have prevented aberrant protein synthesis upon invasion of the introns into protein-coding genes. The emergence of introns in genes would have made the eukaryotic lineage dependent on the spliceosome.

Numerous proteins were added to the spliceosomal core during eukaryogenesis. Many of these are clearly derived from proteins that already had an RNA-binding function [10, 86, 93]. Coincidental interactions with these proteins could have caused presuppression and subsequent dependence, increasing the complexity of the spliceosome without a clear benefit [10, 16, 17]. The expanding repertoire of splicing regulatory proteins would have enabled the decay of clearly defined exon-intron boundary features, leading to dependence as well [10]. In these ways, the present-day spliceosome would have been built up “step by unselected step” [10].

#### An interplay between neutral and adaptive evolution explains spliceosomal evolution

The lack of clear direct benefits of a complex splicing machinery in the early eukaryotes is a strong argument against an adaptive scenario for its evolution. The only plausible direct benefits are compensations for maladaptive features. In light of the small effective population size inferred to have been present during eukaryogenesis based on the fixation of introns [23, 38] or paralogous genes [117], or on the proposed early mitochondrial endosymbiosis event [117–119], this is definitely a possibility. However, a neutral scenario in which these features were tolerated by a more complex spliceosome remains more likely, because a maladaptive intermediate stage does not need to be invoked. Other advantages of spliceosomal introns and concomitantly the spliceosome, like enabling alternative splicing and fine-tuning, work on the long term. These are fully exploited only in multicellular eukaryotes, making it therefore unlikely that this system has evolved for this particular purpose. The small effective population sizes before LECA, and in animals and plants seem to be largely responsible for the drastic increases in complexity of the spliceosome. A role of adaptive processes is not excluded and likely has played a role in certain interactions, but for each new feature the null-hypothesis of neutral, random evolution should convincingly be disproven [114]. Natural selection has definitely played a role in the simplification of introns and the splicing machinery that can be observed in multiple lineages. The selective pressure for streamlining that characterises organisms like yeast and *Giardia* has resulted in a significant loss of introns and spliceosomal components [22, 110, 113]. Clearly, complexification in this process is not truly irremediable and can be overcome by natural selection.

The scenario we infer corresponds to a biphasic pattern of evolution, in which a short explosive innovation phase is followed by a much longer reductive phase [20, 21]. Most of the complexity of the spliceosome emerged during eukaryogenesis. Subsequently, its complexity stabilised or decreased in multiple lineages. However, in the lineages leading to plants and animals, and within the animals the lineage leading to the vertebrates, additional periods of rising complexity took place. Most of the machine’s complexity does not seem to evolve gradually at a somewhat constant rate, but instead in rapid bursts. This alternation of periods of increasing and decreasing complexity has also been described for many other processes [20]. Although often observed, a biphasic pattern does not offer an explanation per se. One potential explanation for these patterns that has been put forward is that complex machines arising through e.g. constructive neutral forces can in subsequent evolutionary time provide an advantage in terms of adaptation in surviving lineages. This explanation has been argued for as a special case of multilevel selection [120, 121] and biphasic genome evolution is one of the most striking outcomes of computational modelling of the interplay between network and genome evolution [21].

## Conclusions

The spliceosome is a complex molecular machine that arose during eukaryogenesis and removes introns from pre-mRNAs, which is required to prevent the production of aberrant proteins. The spliceosome consists of five snRNPs, each comprised of an snRNA and proteins, and additional proteins. There is ample evidence that both the spliceosomal core and the spliceosomal introns originated from self-splicing group II introns, which are widely believed to have been transferred from the mitochondrial endosymbionts to the host DNA. The snRNAs, at least U2, U5 and U6, are likely derived from fragmented group II introns and the U5-snRNP-specific protein Prp8 evolved from the IEP of these introns. Sm proteins, helicases and other proteins were at some point recruited to the spliceosomal core. This addition and the extensive expansion of especially Sm proteins and helicases have drastically increased the complexity of the spliceosome during eukaryogenesis. Apparently, all group II introns in the nucleus were either lost or converted to spliceosomal introns before LECA. During eukaryotic evolution a pronounced increase in spliceosomal complexity occurred in plants and animals, which mainly involved the regulatory proteins. In other lineages the spliceosome simplified, with U2 and U5 snRNP proteins being the least affected, and concomitantly the number of introns decreased.

The spliceosome-like machineries involved in group II intron splicing in some eukaryotic plastids and mitochondria could be an interesting model for the evolution of *trans*-splicing complexes from self-splicing group II introns, as they are less complex and have evolved more recently. Splicing facilitated by general maturases and other protein factors in plant organelles [122] and by an RNP complex comprising a *trans*-acting RNA and protein factors in the plastids of the green alga *Chlamydomonas reinhardtii* [67], and suggested RNP complexes for excising so-called group III introns in the plastids of the excavate *Euglena* [39] are interesting examples of recurrent evolution. These might shed more light on the origin of the spliceosome.

The spliceosome is one of the most complex machines that emerged during eukaryogenesis. Other complex features that originated in the eukaryotic lineage are for example the nuclear pore complex, an elaborate endomembrane system, the RNA interference machinery and the kinetochore [123–126], to name a few. Moreover, multiple machineries inherited from the prokaryotic ancestors increased in complexity, like the ribosome, proteasome and exosome [10, 19, 90]. These examples underscore the contribution of gene duplications to increased machine complexity [19, 90]. It is tempting to speculate that the vast expansion of protein families reflects whole-genome duplications or hybridisation events, perhaps in syncytial early eukaryotes [119]. The importance of neutral processes in the evolution of one of the most complex machines suggests that neutral evolution has contributed significantly to the complexity of other less complex machines as well. A profound reconsideration of the evolutionary forces that shaped these complexes is therefore desired, in which neutral processes should be considered as null-hypothesis.

## Reviewers’ comments

### Reviewer’s report 1: W. Ford Doolittle, Dalhousie University

Reviewer comments:

There is much detailed information here about the evolutionary history and likely prokaryotic origins of many components of the eukaryotic spliceosome. I assume that it is up-to-date and correct. We seem now to know quite a bit about this, and have little doubt that the last eukaryotic common ancestor (LECA) already had a modern-type splicing apparatus and many introns requiring its services (see the author’s reference 38, and Rogozin et al., 2012, Biol Direct 7:11). Nobody now seems to question the notion first mooted by Cavalier-Smith (1991, Trends in Genetics 7:145–148), that introns entered eukaryotic nuclear genomes as transfers of group II introns from the alpha-proteobacterial endosymbionts that became mitochondria, and, as RNAs, broke down into something like the “five easy pieces” described by Sharp (authors’ reference 66). My “introns early” hypothesis (1978, Nature 272: 581–582), appealing as it might have appeared in the late 1970s, is dead and buried.

Authors’ response: *We thank the reviewer for the references he put forward on the historical embedding of this topic and we have added these, if not present yet, in the main manuscript. Although the alphaproteobacterial ancestry (* *via* *the endosymbiont) of the spliceosomal introns is widely believed, we would like to caution that is based on circumstantial evidence. Alternative scenarios in which group II introns were already present in the Asgard-related host before the endosymbiosis event or were transferred from another donor after this event are also plausible. The inferred HGT events of organellar group II introns among organelles, between different eukaryotic groups and between bacteria and eukaryotes* [39, 79] *weaken the link between mitochondrial group II introns and spliceosomal introns, as their presence in LECA’s mitochondria is not evident. This does not preclude the proposed effect of the mitochondrial endosymbionts on the available amount of energy and effective population size, which may have resulted in massive intron proliferation and the origin of the complex spliceosome.*

But there are many questions still to be answered, I think. For instance, why, if invasion of the nuclear genome by Group II introns from mitochondria happened before LECA, and eukaryotic nuclear genomes provide a wealth of targets (non-protein coding regions) where it could happen again, has it not? Inferred genetic and physiological barriers seem (Truong et al. 2015, PLoS Genet 11: e1005422) too weak to explain total absence.

Authors’ response: *This is indeed an important remaining question and we have included some additional sentences on this aspect in the manuscript. We agree that the proposed gene expression problems and lower Mg* ^*2+*^ *concentration do not provide a satisfying explanation for the exclusion of group II introns from the nuclear genome. Additional experiments, also in other eukaryotes, and a closer inspection of relatively recent transfers of group II introns to the nuclear genome, such as described in* [52]*, might elucidate this enigma in the future.*

Another question worth pondering: it is relatively easy to imagine how selective and neutral processes could have given rise to the remarkable complexity of the spliceosome. Indeed, I particularly like how this paper gives credence to the latter, especially what has been called “Constructive Neutral Evolution” (authors’ references 9 and 15). Much harder to imagine is how a complex spliceosome, once it has become essential to the expression of most of the genes in a genome, could ever be simplified. And yet it has been, several times. This may be no more amazing than other instances in which what seem to be wired-in fundamental processes and structures can be completely dispensed with or radically transformed. Selection might be not nearly so important as we Darwinists want to believe, either in the building or the dismantling of complexity (author’s reference 18).

Finally, and to me most interestingly, how can we combine multi-level selection theory with reasoning about introns as adaptations (Doolittle, 1987, Cold Spr Hbr Symp Quant Biol 52: 907–913)? It may well be that multicellular eukaryotes of a certain type (us, for instance) have gained considerable evolvability (and consequent diversity) from having alternatively spliceable introns. But clearly, introns were not added to the genome of LECA so that more than a billion years later this advantage could be realized. Authors are (although too circumspectly in my opinion) down on such teleological rationalizing, but might we imagine such evolvability to be an adaptation at some much higher level (clades above species, Doolittle 2017; Phil Sci 84: 275–295)?

Authors’ response: *We did not mean to neglect or downplay the importance of multi-level selection and evolvability in the evolution of the complex nature of the spliceosome and introns. We have added a short discussion of this aspect at the end of “An interplay between neutral and adaptive evolution explains spliceosomal evolution” when we discuss the biphasic model.*

### Reviewer’s report 2: Eugene V. Koonin, National Center for Biotechnology Information (NCBI)

Reviewer comments:

In this review article, Vosseberg and Snel discuss the origin of the spliceosome that was initiated by the domestication of bacterial self-splicing introns. It is a subject of obvious importance and interest, and a long-standing, hard problem in evolutionary biology. The difficulty of the problem stems from the appearance of “irreducible” complexity: the most primitive eukaryotes we are aware of already have a (more or less) full-fledged spliceosome, with the implication that such was also the case for the LECA. Actually, as the authors emphasize, LECA most likely possessed both known types of spliceosomes, U12 and U2. The spliceosome is one of the best showcases for the evolution of eukaryotic cellular complexity because there can be no direct ancestors of the spliceosome in prokaryotes given the non-existence of splicing other than that of self-splicing introns. And, indeed, the authors summarize the relevant information and make a compelling case for the origin of both the spliceosome and the spliceosomal introns themselves from Group II self-splicing introns. In the discussion of the subsequent evolution of the splicesomes, the authors make a good case for a constructive neutral evolution scenario. I fully agree that, in the least, constructive neutral evolution is the appropriate null hypothesis for the evolution of the spliceosome and other complex eukaryotic features. The authors make a very interesting point about the relatively simple, spliceosome-like complexes that are involved in splicing of Group II introns in organelles. These are not ancestral but their evolution might recapitulate that of the spliceosome, so analysis of such complexes indeed might illuminate spliceosome evolution. On the whole, this is a very useful, interesting and insightful review, and a good read, too.

Authors’ response: *We thank the reviewer for his excellent summary and appreciate the constructive feedback he has provided in his report.*

I do not have particularly serious criticisms of this article. I find the discussion of the potential of phylogenetic analysis of the IEP and Prp8 (lines 230–237) to be rather disingenuous. I agree with the authors that such analysis is unlikely to be particularly informative. However, it seems to me that one should either try and actually do it or drop this line of discussion altogether.

Authors’ response: *We apologize that our initial wording could be seen as disingenuous and we have decided to remove it.*

In the discussion of the evolution of Lsm/Sm proteins, the following “A small number of introns are even shared between certain Lsm-Sm pairs. This is not trivial, as it implies that splicing could already take place before the diversification of the Sm family in eukaryotes” (lines 272–274) is indeed a non-trivial observation, and I think additional details are needed for the reader to be able to assess its validity and impact.

Authors’ response: *We have added more details on these findings and discussed these in light of the low number of shared introns for paralogues originating from duplications during eukaryogenesis and the high number of shared introns for orthologues present in LECA. Upon mapping the location of introns onto the alignments of Lsm/Sm proteins in 22 eukaryotic species, Veretnik* et al. [90] *made the striking observation that for some pairs, and also between certain pairs, at the same position in the alignment an intron was located in multiple species for each paralogue, suggesting that this intron was already present before the duplication event. These findings have two major implications: 1) before the diversification of these proteins, which are essential for present-day splicing, splicing already took place; 2) these gene duplications likely did not occur early during eukaryogenesis.*

For a review article, the current manuscript seems to be somewhat insufficiently referenced. In many case, the authors rely on previous reviews where several original references would do better. I will not suggest a full list of references to add and will only mention two: Along with ref. 96, the following should be cited: López-García P, Moreira D. Selective forces for the origin of the eukaryotic nucleus. Bioessays. 2006 May;28(5):525–33. The absence of this reference which presents a comprehensive reconstruction of intron gain and loss in eukaryotic evolution is surprising: Csuros M, Rogozin IB, Koonin EV. A detailed history of intron-rich eukaryotic ancestors inferred from a global survey of 100 complete genomes. PLoS Comput Biol. 2011 Sep;7(9):e1002150.

Authors’ response: *Upon a critical reassessment of the references we have added 28 references to primary papers throughout the paper and we believe that the references are now more balanced. Initially, we did not include a discussion on the number of introns in LECA, but we agree that this was an omission and therefore added a small paragraph about this in the section "LECA’s spliceosome", including the latter reference. We did not discuss ref.* *96* *in light of the hypothesis on the origin of the nucleus, but for the scenario they propose for the origin of the spliceosome. In the revised version of this review we shortly mention the origin of the nucleus in the “Adaptive model” section, referring to both papers.*

Further, in my view, there are too few figures in the article, and those included are too crude schematics. It would be good to show a better comparison between elements of self-splicing introns and snRNAs, and perhaps, a complete general scenario for the evolution of the spliceosome.

Authors’ response: *Figure* *1* *was drawn as a schematic figure on purpose, to immediately appreciate the similarities between group II and spliceosomal introns. Excellent figures with more details have been made before (*e.g.*, Fig. 5 in* [39]*). We have added more details in Fig.* *1* *by depicting also the RNA structures that are not similar and indicating that not the entire proteins are homologous. As suggested by the reviewer, we have also made a new figure (Fig.* *3* *) depicting the general scenario we propose with regards to spliceosome evolution pre- and post-LECA.*

### Reviewer’s report 3: Vivek Anantharaman, NCBI

Reviewer comments:

The authors have written a review of the evolution of the complexity of the spiceosomal machinery. They have touched on the various components of the spliceosome and their evolution. While there are many detailed discussions of this material available, this is a useful update summarizing the various ideas. Hence I find the review satisfactory and worthy of being published as is.

The authors have presented a review of the prevalent ideas in the subject satisfactorily. I do not have any major recommendations.

In pg 9–10 the authors discuss the loss of RT activity in Prp8 and point to a 2015 article. The inactive Prp8 and its possible link to Group II intron and spliceosomal evolution has been discussed in a much earlier 2012 paper (pmid: 22919680) by our group.

Authors’ response: *The reference to the 2015 paper was actually for the last part of this sentence. We have added references to the suggested paper and another paper when discussing the loss of RT activity.*

## Abbreviations

EGT: Endosymbiotic gene transfer
HGT: Horizontal gene transfer
hnRNP: Heterogeneous nuclear ribonucleoprotein
IEP: Intron-encoded protein
LECA: Last eukaryotic common ancestor
Prp8: Pre-mRNA processing protein 8
RNP: Ribonucleoprotein
RT: Reverse transcriptase
snRNA: Small nuclear RNA
snRNP: Small nuclear ribonucleoprotein
SR protein: Serine/arginine-rich protein
